# Educating tomorrow's donors: a high school initiative to promote blood and stem cell donation through stem education

**DOI:** 10.1186/s12909-025-07547-3

**Published:** 2025-07-01

**Authors:** Katia Mareschi, Alessia Giovanna Santa Banche Niclot, Elena Marini, Camilla Francesca Proto, Federico Divincenzo, Claudia Peirolo, Giulia Zucchetti, Franca Fagioli

**Affiliations:** 1 Department of Public Health and Paediatrics, University of Turin, Piazza Polonia 94, Turin, 10126 Italy; 2 Paediatric Onco-Haematology Division, Regina Margherita Childrens’ Hospital, City of Health and Science of Turin, Turin, 10126 Italy

**Keywords:** Education, Donation, Hematopoietic stem cell transplantation, High school, Adolescents

## Abstract

**Background:**

Hematopoietic stem cell transplantation (HSCT) is a life-saving procedure for pediatric patients with hematologic malignancies, genetic disorders, and other serious conditions. However, its success critically depends on the availability of compatible donors, which remains a challenge—especially in ethnically homogeneous populations. Raising awareness among adolescents may help expand donor registries and promote civic engagement in public health.

**Methods:**

This study, conducted by the Pediatric Onco-Hematology team at an Italian University, aimed to bridge the gap in donor awareness among adolescents by implementing an educational intervention to increase knowledge about blood diseases, stem cell donation, and the donor registration process. The intervention, which targeted high school students, sought to foster informed decision-making and encourage future blood and stem cell donors.

The program involved 900 students (aged 16–18) from 12 scientific-track schools. Designed in collaboration with school staff and aligned with national curricular guidelines in biology and civic education, the intervention included 2-h scientific seminars and observation-based visits to a Children’s Hospital transplant center. A 10-item post-intervention Likert-scale questionnaire assessed seminar clarity, student engagement, understanding of donation processes, satisfaction with the presenter, and willingness to register as donors. Two open-ended questions invited qualitative feedback. Data were analyzed using descriptive statistics and thematic analysis.

**Results:**

A total of 346 students completed the survey. Quantitative data showed high levels of satisfaction and increased awareness of blood and stem cell donation. Many students expressed interest in future donor registration and biomedical career paths. Thematic analysis of open-ended responses revealed strong appreciation for real-world laboratory exposure, clarity of scientific content, and the motivational impact of learning about donation. No pre-intervention survey was conducted, which limits comparisons.

**Conclusions:**

The program effectively promoted awareness of blood and stem cell donation among adolescents and stimulated interest in STEM-related careers. Given its structured and adaptable format, this model could be implemented in other educational contexts to enhance health literacy and support donor recruitment efforts. Future editions will include pre-post evaluation and longitudinal tracking to assess sustained impact.

**Supplementary Information:**

The online version contains supplementary material available at 10.1186/s12909-025-07547-3.

## Introduction

Hematopoietic stem cell transplantation (HSCT) represents a therapeutic intervention that is frequently life-saving for a wide range of pediatric conditions, including hematologic malignancies, severe aplastic anemia, and certain genetic disorders [1, 2]. This complex procedure, which involves the infusion of healthy hematopoietic stem cells to restore bone marrow function, offers the potential for cure or significant disease remission in cases where conventional treatments may be insufficient. However, the success of HSCT is highly contingent on the availability of compatible donors, which remains a significant challenge, particularly in pediatric populations where timely and suitable matches are crucial for optimal outcomes [3].

Despite global efforts to increase donor registrations, with over 42 million potential donors worldwide as of 2023 [4], finding compatible donors for pediatric patients continues to be a significant hurdle. In many cases, especially in smaller or ethnically homogeneous populations, the likelihood of finding a match is reduced, underscoring the importance of expanding donor registries and increasing awareness about the critical need for stem cell donations. Educational initiatives targeting adolescents have shown promise in addressing this gap, as they significantly enhance knowledge and attitudes towards stem cell donation ^5,6^. Adolescents represent a unique demographic; not only they have the potential to become future donors, but they are also at a developmental stage where they can absorb information that promotes healthy lifestyles and fosters a mature, responsible civic culture [5, 6].

In this context, STEM (Science, Technology, Engineering, and Mathematics) education in high schools plays a crucial role. It not only fosters critical thinking, problem-solving, and scientific literacy, but also shapes students' future career paths, particularly in health-related fields. Incorporating topics like blood and stem cell donation within STEM curricula provides students with the tools and understanding they need to engage with complex medical issues and public health challenges. Early exposure to biomedical topics through STEM education can inspire students to pursue careers in medicine, research, and healthcare, where their contributions can directly impact public health initiatives such as increasing donor registrations for life-saving procedures like HSCT. By linking public health education to broader STEM goals, students are better prepared to make informed decisions, contributing not only to their personal development but also to the health of their communities [7, 8].

In Italy, however, there is a notable lack of structured educational programs within schools that specifically address the importance of blood and stem cell donation. This gap is particularly concerning given the critical need for increasing donor registrations to support HSCT procedures. Unlike other countries where school-based programs have led to a rise in donor registrations, Italian adolescents often remain uninformed about the importance of stem cell donation and how they can contribute.

To address this gap, this study, conducted by the University of Turin's Pediatric Onco-Hematology team, introduces a novel educational intervention aimed at improving awareness of blood diseases, stem cell donation, and donor registration among high school students in Turin. By integrating these topics into the high school curriculum, the intervention seeks to foster informed decision-making and encourage a new generation of donors who are knowledgeable about the life-saving impact of HSCT.

## Methods

### Study design and participants

This study was conducted over the 2022-2023 and 2023-2024 school years, starting in March 2022. It targeted students aged 16 to 18 from public high schools with a scientific curriculum in Turin and the surrounding areas. The study involved a total of 900 students from 12 different high schools*. Participation in the seminars was open to entire classes within the scientific high school curriculum, selected by the school as part of their local orientation and career guidance program (in Italian we defined them as PCTO*=*Pathways for Transversal Skills and Orientation" framework. The content was aligned with national curricular standards in biology and civic education, ensuring relevance to students’ academic programs.*

### Education proposals

Two main educational activities were designed and implemented as part of the local program for acquiring *transferable skills and career orientation* designed by high schools in collaboration with the orientation office of University of Turin (medicine center) to guide students in their future career or university choices. These activities were developed in collaboration with science professors and school administrative staff as illustrated in Table 1.

**Table 1:** Structure and content of the educational program

Activity	Duration	Format	Target Audience (16–18 y.o.)	Selection Criteria
Scientific Seminar	2 hours	In-person presentation with slides and videos	Entire scientific classes	Selected by school as part of PCTO
Observation-based Clinical Immersion	3 hours	Hospital tour and lab observation	15 students per school	Interest and academic merit

#### Scientific seminars

The seminars, titled "Stem Cells: What We Know, Their Functions, and Their Use in Clinical Practice and Future Potential," were 2-hour sessions conducted by a professional biologist. *Each 2-hour seminar was conducted individually in each of the participating high schools as part of the PCTO program. The format and content were consistent across all 12 institutions.* The sessions included presentations with slides and videos covering a range of topics:

- ◦ Basic stem cell biology
- ◦ Hematological disorders
- ◦ The process and significance of bone marrow transplantation
- ◦ The importance of stem cell donation* supporting HSCT and in general clinical care.*
- ◦ The registration process for becoming a blood and stem cell donor.
- ◦ The use of mesenchymal stem cells and their preclinical applications.

The seminars were designed to be engaging and informative, using multimedia to enhance understanding.

#### Practical experience

A select group of 15 students from each participating school, chosen based on their interest and academic performance, participated in a practical experience. Selection was based on teacher assessment of academic interest, motivation shown in class discussions, and participation in science-related extracurricular activities.

The practical sessions were held at the Transplant and Stem Cell Center of the Regina Margherita Children’s Hospital in Turin. Students observed real-world procedures involving the use of hematopoietic stem cells from donation to infusion. The experience included demonstrations of:

- Molecular biology techniques
- Cell culture maintenance
- Quality control processes
- Clinical procedures related to stem cell transplantation.

For safety reasons, students participated as observers only, allowing them to gain insights into clinical and laboratory practices without direct involvement.

### Data collection

After the educational activities, all participants were asked to complete a 10-question Likert scale survey designed to assess their knowledge, attitudes towards stem cell donation, and overall satisfaction with the educational experience. The survey was administered via QR code displayed at the end of each seminar session, and participation was voluntary. The questionnaire addressed key aspects such as student engagement, clarity of content, effectiveness and approachability of the instructor, and appropriateness of the information presented. It also included questions about the willingness to register as a stem cell donor and the evaluation of the hospital visit. Two open-ended items explored the most valuable aspects of the seminar and suggestions for improvement.

Survey responses were analyzed using descriptive statistics (frequencies and percentages), without inferential analyses due to the exploratory nature of the study. A full English version of the questionnaire is provided as supplementary material.

The data summarized in Table 2 refer to post-intervention responses and represent only positive feedback—specifically, answers marked as Agree or Strongly Agree on the Likert scale. For example, "Appreciation of the activity" aggregates favorable responses related to student engagement, clarity of the content, and satisfaction with the presenter.

**Table 2:** Summary of positive feedback from post-intervention questionnaire

Section	Parameter	N	Percentage
General	Total number of students involved	900	—
	Students who completed the post-intervention questionnaire	346	—
	Number of participating institutes	12	—
Theoretical Lessons	Appreciation of the activity	328	95%
Blood Donation	Students who answered 'Absolutely yes' to blood donation	92	26.5%
	Students who answered 'Maybe' to blood donation	144	41.6%
	Students who answered 'I don't know' to blood donation	88	25.4%
	Students who answered 'Absolutely not' to blood donation	22	6.4%
ADMO registry subscription	Students who answered 'Absolutely yes' to joining Bone Marrow Donor Registry (ADMO)	64	18.5%
	Students who answered 'Maybe' to joining Bone Marrow Donor Registry (ADMO)	180	52.0%
	Students who answered 'I don't know' to joining Bone Marrow Donor Registry (ADMO)	84	24.3%
	Students who answered 'No' to joining Bone Marrow Donor Registry (ADMO)	18	5.2%
Practical Activities * students involved in these activities	Validity of the proposal	183/183*	100%
	Interest in the activity	170/183*	93%
	Interest in enrolling in a biomedical faculty	74/183*	40.7%
	Interest in pursuing university research	84/183*	46.2%

To complement the descriptive statistical analysis, an exploratory thematic analysis of open-ended responses was conducted. The initial categorization of recurring themes was supported by ChatGPT (OpenAI), an AI-based language model, used as a tool to assist with pattern recognition and organization of qualitative data. All themes and selected representative quotes were subsequently reviewed and validated by the research team to ensure interpretative consistency and relevance.

## Results

Of the 900 students involved in the educational program, 346 completed the post-intervention survey. Despite the voluntary nature of participation, the responding group was sufficiently large and diverse to be considered representative of the overall student population involved. The questionnaire responses reflect students’ perceptions of the seminars and the optional laboratory experience at the Children’s Hospital.

The results of the study are summarized in Table 2 which reports key indicators related to student participation, their appreciation of the educational activities, and the potential impact on their future choices regarding biomedical studies and donor registration.

Feedback from participants highlighted the educational value of the hospital experience and the importance of raising awareness about blood and stem cell donation. Students appreciated the practical sessions because they had the opportunity to deepen their understanding of topics related to their education and explore possible university paths in the medical and biological fields. They were sensitized to the importance of blood and stem cell donation, witnessing firsthand how these donations can have life-saving effects, particularly for individuals their own age suffering from severe diseases like leukemia.

Additionally, the program helped connect the content of the seminars and practical experiences to broader STEM (Science, Technology, Engineering, and Mathematics) disciplines. The exposure to real-world biomedical applications not only heightened interest in health sciences but also demonstrated how knowledge from biology, technology, and laboratory techniques plays a crucial role in medical research and public health. This connection was particularly meaningful for students who had not previously considered pursuing a career in STEM fields but were now more inclined to explore opportunities in biomedical research, medicine, and other scientific disciplines.

Positive feedback was also received from teaching staff involved in the program, although no formal evaluation questionnaires were provided. In particular, teachers reported directly to the programme coordinator their appreciation for the real-world exposure offered by the hospital visit, and noted increased student engagement in related classroom discussions. The educators recognized the value of linking theoretical knowledge from the classroom to practical, real-world applications in a clinical setting.

Although the initial survey was primarily quantitative and did not include structured qualitative interviews, two open-ended questions invited students to describe the most valuable aspects of the seminar and to suggest improvements. Table 3 summarizes a qualitative thematic coding of the open-ended responses from high school students. The 'Other/Not Classifiable' category includes responses that were too brief, vague, or unrelated to the core topics addressed, and thus could not be reliably categorized within the thematic framework. Each response was reviewed for recurring patterns and grouped under dominant themes. Quotes were selected for clarity and representativeness. Thematic analysis revealed recurrent topics such as the value of laboratory experiences, increased awareness of the importance of blood and stem cell donation, the usefulness of the information provided, and suggestions for improving engagement (e.g., more interactivity or integration of videos). The most frequently mentioned theme was the observation-based laboratorial immersion, followed by comments on donation awareness and the clarity of scientific explanations. Several students explicitly mentioned that they were unaware of the possibility and importance of donating stem cells before the seminar. Others indicated that the initiative had inspired them to consider biomedical careers.

**Table 3:** Thematic analysis of open-ended student responses

Theme	Frequency	Representative Quotes
Laboratory Experiences	54	“I was most struck by the cell culture and seeing how they are handled.” “Visiting the labs and seeing the equipment in person was very impactful.”
Suggestions / Improvements	37	“Maybe more videos during the lecture would help avoid fatigue.” “I would have spread out the hours of explanation instead of putting everything in one day.”
Donation (blood / stem cells)	12	“I realized how easy and important it is to donate, and the awareness it gave me.” “I didn’t know stem cells could be used to cure so many diseases.”
Informational Usefulness	11	“The seminar helped me understand complex topics I hadn’t encountered before.”
Surprise or New Discoveries	3	“I had no idea stem cells could be used in so many ways.”
Other / Not Classifiable	229	“Responses too generic or unrelated to categorize clearly.”

One student commented:

*"In my opinion, the experience at the Regina Margherita Hospital was very educational and useful, especially in light of my future university path. Studying the theory (about blood and stem cells) and then seeing it applied in practice doesn't happen often, especially in a school setting. I also believe it is essential to raise awareness among 18-year-old students about blood donation and its consequences."*

Another student added:

*"Although I did not participate in the more 'practical' part of this experience, I can evaluate the lectures I attended: they were quite interesting, both in providing an idea of the career opportunities that science faculties can offer and in informing and raising awareness about existing pathologies that should be seriously considered. I particularly appreciated the information about blood and bone marrow donation, both of which I intend to pursue as soon as possible. In conclusion, even though I am not leaning toward scientific studies, I found this PCTO experience enriching."*

These reflections illustrate the widespread appreciation of the educational activities and indicate how such experiences can serve as a foundation for future educational and career choices. Beyond raising awareness of blood and stem cell donation, the program encouraged students to consider STEM-related careers in biology, medicine, and biomedical research. By linking public health issues with broader scientific principles, the program contributed to fostering a generation more inclined to pursue STEM disciplines and make informed decisions that could positively impact public health.

## Discussion

### Program impact

Our study addresses a gap in the literature on educational initiatives in Italian high schools aimed at enhancing awareness of blood and stem cell donation. Few studies have explored the impact of these programs on adolescents, underscoring the need for targeted research. While similar programs have been implemented in other countries [9–12] with positive outcomes, the Italian context has largely remained unexplored. Our intervention, which combines scientific seminars with observation-based clinical immersions, represents a novel approach to engaging adolescents in this important public health issue.

A key strength of our program is its scientifically rigorous content, tailored to a demographic crucial for the future of blood and stem cell donation. Adolescents are at a formative stage where they shape their beliefs and attitudes, making this an opportune moment to introduce concepts related to donation and public health responsibility [13, 14]. By providing comprehensive education on the importance and implications of stem cell donation, our program not only increases knowledge but also fosters a generation more likely to participate in life-saving medical procedures. This is particularly impactful because students learn that their decision to become donors could one day save the lives of young people their own age who are suffering from serious conditions such as leukemia or other blood diseases [15].

### Comparison with similar initiative

Similar findings were observed in an educational intervention study. While the educational model reference involved organ donation, which differs fundamentally from hematopoietic stem cell (HSC) donation as the latter is renewable and minimally invasive, study on solid organ donation among medical students, where significant improvements in knowledge and attitudes towards organ donation were recorded after an educational session. Prior to the intervention, only 27.10% of students knew about the organs that could be donated, but this increased to 80% after the educational intervention. The study demonstrated that targeted education is effective in significantly improving students' understanding and awareness of organ donation and highlighted the need for structured educational interventions to motivate and inform students about such critical public health topics [12]. Like our findings, this study reinforces the idea that educational interventions can bridge the gap between knowledge and positive behavioural intentions, leading to a more informed and motivated potential donor base. Both studies underscore the critical role of education in shaping students' beliefs and encouraging participation in life-saving health interventions.

### Role of educators

Teachers and school staff should be equipped with the knowledge and skills necessary to effectively deliver these programs. Collaborations with healthcare professionals, especially those specialized in hematology and stem cell transplantation, could further enhance the quality and accuracy of the information provided, ensuring that students receive the most current and scientifically sound knowledge.

### Implications for STEM and public health education

Another important aspect of our study is the emphasis on key educational frameworks that promote critical thinking, problem-solving, and scientific literacy, all essential goals in STEM education. The seminars and practical experiences were designed not only to raise awareness about blood and stem cell donation but also to engage students in analytical thinking and hands-on learning.

By linking the program to broader STEM career pathways, we highlight the potential of such initiatives to increase student interest in fields like biomedical research. The high percentage of students (46.2%) who expressed an interest in pursuing university studies in biomedical sciences reinforces the idea that these programs can significantly impact STEM engagement and career development, aligning with findings from other studies in STEM education [16, 17].

Similarly, the 18.5% of respondents who expressed a clear willingness to join the Bone Marrow Donor Registry (ADMO), together with the 52.0% who reported being open to the possibility (“Maybe”), and the 24.3% who were uncertain (“I don't know”), suggest that the intervention helped initiate reflection on civic responsibility and donation awareness. The combined proportion of students who did not reject the idea of registration (94.8%) highlights the program’s potential to influence future attitudes. This openness—despite the complexity of the topic and the students’ young age—underlines the need for follow-up activities to support decision-making and reinforce key messages over time, even if behavioral change cannot yet be confirmed. In addition to stem cell donation, students’ responses regarding blood donation further underscore the program’s civic impact. Notably, 26.5% of students declared they would “absolutely” donate blood, and 41.6% responded “maybe,” while only 6.4% firmly rejected the idea. These findings indicate that a large majority of participants were either open to or seriously considering blood donation. Given that blood donation is more immediate and accessible to 18-year-olds compared to stem cell donation, these attitudes suggest that the intervention may have also fostered a concrete sense of agency and contribution to public health. This highlights the importance of including information on all types of donation within school-based education and calls for targeted follow-up campaigns as students reach legal donor age.

Moreover, our findings suggest that by integrating these real-world applications into the curriculum, students are more likely to develop positive attitudes toward public health issues and consider career paths they may not have previously explored. The connection between practical STEM activities and increased interest in fields such as biomedical research further emphasizes the need for interdisciplinary educational programs that not only teach scientific content but also provide context for real-world applications. This aligns with findings in STEM education research, where students' participation in interdisciplinary STEM activities was shown to enhance their views on education and future career opportunities.

To maximize the impact of such initiatives, integrating donation awareness programs into the broader high school curriculum—particularly in biology or health education courses—would ensure that all students, not just those who opt into extracurricular activities, receive essential information about donation. Embedding this content into the standard curriculum could create a more inclusive approach, reaching a wider audience and normalizing the conversation around donation from an early age [18].

Our study demonstrates the effectiveness of well-structured educational programs in high schools, particularly those led by trained professionals who can distill complex medical information into accessible and engaging content. The significant increase in new blood donors aged 18–25 in the Turin registry [19, 20] observed from 2021 to 2023 suggests that our intervention may have contributed to this rise in donor registrations, although further research is needed to establish a causal relationship. Providing students with observation-based laboratorial immersions, such as opportunities to observe real-world applications of what they learn in the classroom, as demonstrated in our program, can significantly enhance their understanding and retention of the material. Such experiences make the concept of donation more tangible and relatable, reinforcing the importance of their potential role as future donors.

The study could be further strengthened by discussing the long-term impact of these educational interventions. One important question is whether the program aims to track students over time to determine how many actually register as donors or pursue careers in biomedical research. A longitudinal aspect—where participants are followed for several years—would provide valuable data on how early exposure to donation and medical science translates into sustained involvement in these areas. By tracking participants, we could assess whether the increased awareness and positive attitudes towards stem cell donation, as demonstrated in the short term, lead to long-term behavioral changes such as joining the donor registry or choosing careers in STEM fields. Adding this longitudinal component would make the study more compelling for an audience interested in sustained educational outcomes and public health contributions.

In addition, this program has the potential to serve as a model for other regions or countries seeking to integrate similar topics into high school STEM curricula. By demonstrating the success of this initiative, educational policymakers could be encouraged to adopt these types of programs to foster both public health awareness and STEM education. Expanding the program beyond Turin to other regions of Italy and potentially internationally would ensure that a broader demographic of students benefits from early exposure to critical public health issues such as blood and stem cell donation.

### Limitations and future directions

This study has several limitations. First, the absence of a pre-intervention assessment limits the ability to measure knowledge gain. Second, the selection of students for the clinical immersion was based on teacher judgment, potentially introducing selection bias. Third, the lack of disaggregated demographic data (e.g., gender or school type) limits subgroup analysis. Lastly, we cannot infer a direct causal relationship between this intervention and the observed increase in donor registrations in the region.

Additionally, in some schools, technical difficulties (e.g., lack of internet connection or insufficient time) may have limited the number of responses collected or prevented the survey from being administered entirely.

Future iterations of the program will seek to incorporate pre- and post-intervention assessments, structured feedback from teachers, and long-term follow-up to evaluate the sustained impact on students’ behaviors and career choices. In future research, we plan to implement a longitudinal design with follow-up surveys at 12 months after the intervention. These will assess the retention of knowledge, evolution of attitudes toward donation, and actual registration behaviors once participants reach legal eligibility. Additionally, we intend to explore correlations between willingness to donate and prosocial traits—such as empathy, civic responsibility, and altruism—which may be enhanced through targeted educational interventions. By examining the emotional and behavioral dimensions of donation-related decisions, we aim to identify modifiable factors that can be leveraged to promote long-term civic engagement and health-related altruism. This longitudinal component will provide insight into whether short-term awareness translates into long-term behavior change. Collecting longitudinal data would strengthen the evidence base and inform broader educational and public health strategies.

Moreover, the structure and outcomes of this program suggest that it could serve as a model for future public health education initiatives in secondary schools. Its interdisciplinary approach—linking civic engagement, scientific literacy, and real-world biomedical exposure—makes it a promising and scalable tool for promoting health awareness among adolescents.

## Conclusions

Our program effectively bridges academic learning with real-world applications, emphasizing the need to integrate donation awareness into high school curricula. By combining seminars and observation-based clinical immersions., the program significantly increased students' knowledge and attitudes toward donation, emphasizing how their contributions could save lives, particularly of young people their own age. The initiative also promoted critical thinking and sparked interest in biomedical careers, with nearly half of the students expressing a desire to pursue further studies in this field. Expanding this program to other regions of Italy could help increase donor registrations and foster a culture of donation.

Given its structure and preliminary results, this program may also serve as a scalable and adaptable model for public health education in secondary schools. By linking scientific content with civic engagement and real-world biomedical exposure, it offers a valuable framework for promoting health literacy and donation awareness among adolescents.

Tailoring the initiative to fit local educational contexts and conducting longitudinal studies would provide valuable insights into its long-term impact. Despite some limitations, our findings suggest that this educational model is effective in raising awareness and promoting reflection among adolescents, and may serve as a valuable framework for connecting public health with STEM education.

## Supplementary Information

Supplementary Material 1.

## Data Availability

The data consist of summaries from anonymous questionnaires completed by underage students. Due to privacy regulations, the raw data cannot be made publicly available. However, a sample of the questionnaire used and translated in English is provided as supplementary material.

## References

[1] Myers KC, Davies SM. Hematopoietic stem cell transplantation for bone marrow failure syndromes in children. Biol Blood Marrow Transplant. 2009;15(3):279–92. 19203719 10.1016/j.bbmt.2008.11.037

[2] Okamoto Y, Nakazawa Y, Inoue M, Watanabe K, Goto H, Yoshida N, et al. Hematopoietic stem cell transplantation in children and adolescents with nonremission acute lymphoblastic leukemia. Pediatr Blood Cancer. 2020;67(12):e28732. 32960494 10.1002/pbc.28732

[3] Alsalamah S, Alramyan R, Alakel R, Altheyeb F, Alrashed R, Masuadi E, et al. The outcome and complications of total parenteral nutrition in pediatric hematopoietic stem cell transplantation. Pediatr Transplant. 2022;26(3):e14198. 34811859 10.1111/petr.14198

[4] WMDA. [citato 20 giugno 2024]. WMDA | Matching Donors | Serving Patients. Disponibile su: https://wmda.info/.

[5] Kaya Z, Gültekin KE, Demirtaş OK, Karadeniz D, Çalapkulu Y, Tap Ö. Effects of targeted education for first-year university students on knowledge and attitudes about stem cell transplantation and donation. Exp Clin Transplant. 2015;13(1):76–81. 25275861 10.6002/ect.2014.0023

[6] Zito E, Alfieri S, Marconi M, Saturni V, Cremonesi G. Adolescents and blood donation: motivations, hurdles and possible recruitment strategies. Blood Transfus. 2012;10(1):45–58. 22249786 10.2450/2011.0090-10 PMC3258989

[7] Dost G. Students’ perspectives on the ‘STEM belonging’ concept at A-level, undergraduate, and postgraduate levels: an examination of gender and ethnicity in student descriptions. Int J STEM Educ. 2024;11(1):12.

[8] Li Y, Wang K, Xiao Y, Froyd JE. Research and trends in STEM education: a systematic review of journal publications. Int J STEM Educ. 2020;7(1):11.

[9] NHS Blood Donation. Schools are now teaching young people how to donate and save lives. Disponibile su: https://www.blood.co.uk/news-and-campaigns/news-and-statements/schools-are-now-teaching-young-people-how-to-donate-and-save-lives/. Citato 14 ottobre 2024.

[10] Felts WM, Glascoff MA. Education for blood donation as an avenue for school and community cooperation. J Sch Health. 1990;60(3):103–5. 2319749 10.1111/j.1746-1561.1990.tb05411.x

[11] France CR, France JL, Ysidron DW, Martin CD, Duffy L, Kessler DA, et al. Blood donation motivators and barriers reported by young, first-time whole blood donors: examining the association of reported motivators and barriers with subsequent donation behavior and potential sex, race, and ethnic group differences. Transfusion. 2022;62(12):2539–54. 36281204 10.1111/trf.17162 PMC9742189

[12] Byrne M, Stainer B, Symington M, Leighton J, Jackson H, Singhal N, et al. School education to increase organ donation and awareness of issues in transplantation in the UK. Pediatr Transplant. 2019;23(5):e13492. 31157497 10.1111/petr.13492

[13] Mustanski BS, Viken RJ, Kaprio J, Pulkkinen L, Rose RJ. Genetic and environmental influences on pubertal development: longitudinal data from Finnish twins at ages 11 and 14. Dev Psychol. 2004;40(6):1188–98. 15535766 10.1037/0012-1649.40.6.1188

[14] National Academies of Sciences E, Division H and M, Education D of B and SS and, Board on Children Y, Applications C on the N and S behavioral S of AD and I, Backes EP, et al. Adolescent Development. In: The Promise of Adolescence: Realizing Opportunity for All Youth. National Academies Press (US); 2019 [ 14 ottobre 2024]. Disponibile su: https://www.ncbi.nlm.nih.gov/books/NBK545476/. 31449373

[15] Bloch EM, Mast AE, Josephson CD, Klein HG, Eder AF. Teenage blood donors: are we asking too little and taking too much? Pediatrics. 2017;139(4):e20162955. 28258073 10.1542/peds.2016-2955 PMC5470587

[16] Chen Y, So WWM, Zhu J, Chiu SWK. Stem learning opportunities and career aspirations: the interactive effect of students’ self-concept and perceptions of STEM professionals. Int J STEM Educ. 2024;11(1):1.

[17] Hiğde E, Aktamış H. The effects of STEM activities on students’ STEM career interests, motivation, science process skills, science achievement and views. Think Skills Creat. 2022;43:101000.

[18] OECD. Educating 21st Century Children: Emotional Well-being in the Digital Age [Internet]. Burns T, Gottschalk F, curatori. OECD; 2019 (Educational Research and Innovation). https://www.oecd-ilibrary.org/education/educating-21st-century-children_b7f33425-en

[19] AVIS Torino - Dove e come donare. Disponibile su: http://www.avistorino.it/donazione/diventa-donatore

[20] Fidas Adsp - Donatori Sangue del Piemonte. Disponibile su: https://www.fidasadsp.it/.

[21] Decreto Ministeriale n. 774 del 4 settembre 2019 - Decreto Ministeriale n. 774 del 4 settembre 2019. https://www.mim.gov.it/-/decreto-ministeriale-n-477-del-4-settembre-2019.

