# Supplementary material for: Educating tomorrow's donors: a high school initiative to promote blood and stem cell donation through stem education
Source: BMC Med Educ. 2025 Jul 1;25:962. doi: 10.1186/s12909-025-07547-3 (PMC12220465; doi:10.1186/s12909-025-07547-3)
Supplement: Supplementary file 1 — Supplementary Material 1. [file 12909_2025_7547_MOESM1_ESM.pdf]

Supplementary Material – Full Post-Intervention Student Questionnaire

No.	Question	Response Options (Likert scale 1-5)
1	The level of engagement required by the seminar was appropriate.	Strongly Disagree<br>Disagree<br>Neutral<br>Strongly Agree
2	The instructor was an effective presenter and explainer.	Strongly Disagree<br>Disagree<br>Neutral<br>Strongly Agree
3	The instructor was available and kind.	Strongly Disagree<br>Disagree<br>Neutral<br>Strongly Agree
4	The objectives of the seminar were clear.	Strongly Disagree<br>Disagree<br>Neutral<br>Strongly Agree
5	The content of the seminar was clearly presented.	Strongly Disagree<br>Disagree<br>Neutral<br>Strongly Agree
6	The amount of information provided was appropriate.	Strongly Disagree<br>Disagree<br>Neutral<br>Strongly Agree
7	What aspects of this seminar were most useful or valuable to you?	Open-ended response
8	How would you improve this seminar?	Open-ended response
9	Will you register (or are you already registered) with the Bone Marrow Donor Registry (ADMO)?	Strongly Disagree<br>Disagree<br>Neutral<br>Strongly Agree
10	If you attended the laboratory session at Regina Margherita Hospital, how would you rate it?	Strongly Disagree<br>Disagree<br>Neutral<br>Strongly Agree
